# Case Report: Neuroleptic malignant syndrome in a HIV-positive patient

**DOI:** 10.12688/f1000research.27165.2

**Published:** 2021-11-04

**Authors:** Sibi Joseph, Jerry George, Mongezi Tau, Lourdes de Fatima Ibanez-Valdes, Thozama Dubula, Humberto Foyaca-Sibat

**Affiliations:** 1 Department of Neurology, Walter Sisulu University/Nelson Mandela Academic Hospital, Mthatha, Eastern Cape, 5100, South Africa; 2 Department of Internal Medicine, Walter Sisulu University/Nelson Mandela Academic Hopsital, Mthatha, Eastern Cape, 5100, South Africa

**Keywords:** Neuroleptic Malignant Syndrome, HIV, permanent focal simple motor seizure, elevated creatine kinase, neuroleptic side effects.

## Abstract

We report a 29-year-old, HIV-positive woman being treated with antipsychotic medication for psychosis (Clopixol 200mg intramuscularly monthly, Risperidone 2mg orally daily Haloperidol 2.5mg twice a day), who presented with neuroleptic malignant syndrome. She was also receiving lorazepam and sodium valproate. The patient was referred to our department as she had developed involuntary upper limb movements and simple permanent focal seizure on the lower part of the left hemiface. Clinically the patient had altered consciousness, autonomic dysfunction, and rigidity. Her blood tests showed elevated creatine kinase (1467U/L) but no leucocytosis. We did a thorough workup for other causes of such a presentation. A comprehensive history was taken from the family to exclude other medications used. Her cerebrospinal fluid results were average. Blood tests did not show evidence of infection or other abnormalities. Computed tomography brain was normal. The patient died a few days after the beginning of the attack, which we have also observed  in other HIV-female patients. As far as we know, it is the first report about this comorbidity reported in the medical literature.

## Introduction

Most authors define neuroleptic malignant syndrome (NMS) as a rare, yet life-threatening, idiosyncratic reaction to medications, mostly but not limited to neuroleptic drugs (dopamine receptor antagonists) ^1– 3^. Though fatal, it is potentially a treatable condition ^4^.

NMS was initially described by Delay and colleagues in 1960, who noticed it in patients treated with high-potency antipsychotics ^5, 6^. Though rare, every clinician must bear in mind that this is a significant differential to consider and that it is also a diagnosis of exclusion. One study conducted in 1986 discovered about 500 patients who were on neuroleptics, and about 1.4% clinically had NMS, while one case was fatal since it was not found in time ^1^.

Here we present the case of a young HIV-positive woman whose history was not evident to us initially and had permanent focal seizures on the lower part of the left hemiface and features meeting Leveson’s and Diagnostic and Statistical Manual of Mental Disorders (DSM)-IV criteria for NMS. The patient passed away on the third day of admission. To the best of our knowledge, a case such as this has not been reported in the literature before.

## Case presentation

A 29-year-old woman with a past medical history of chronic psychosis and agitated behaviour presented to the Neurology Outpatient Department of Nelson Mandela Academic Central Hospital in Mthatha, South Africa in June 2020. For the past three months, the patient has been an inpatient at the Level I regional hospital because she presented with a sudden onset of confusion and restless behaviour, including jumping fences and foul language, palpitations, delusions of persecution, and anxiety. She also complained of tachycardia, diaphoresis, and urinary disturbances. The patient received Clopixol (200 mg IM monthly), Risperidone (2 mg PO nocte), Lorazepam (2.5 mg PO nocte), Valproic acid (600 mg PO twice), and (Haloperidol 2.5 mg twice a day) during this hospitalization. Laboratory tests revealed the following: haemoglobin, 11 g/Dl (normal range, 12–15 g/dL); erythrocyte sedimentation rate, 42mm/hr (0–10 mm/hr); creatine kinase, 3049 U/L (20–180 U/L); creatinine, 96 µmol/L (49–90 µmol/l); and alanine transaminase, 47 U/L (7–45 U/L). Cerebrospinal fluid (CSF) was normal.

The patient was admitted to the Neurology Outpatient Department due to altered mental status, permanent partial simple myoclonic seizure on the left lower hemiface, and involuntary upper limb movement. On examination, her blood pressure was labile (ranging from 129/79mmHg to –158/112 mm Hg), and she had tachycardia (119 beats/minute) and mild dehydration. The patient was fully conscious but disorientated to place and time with generalized muscle rigidity characterized by bilateral cogwheel signs at the wrist level and lead pipe signs on both elbows and knees. Bilateral resting tremors of the upper limbs with focal simple myoclonic seizures on the left lower face were observed. She had decreased muscle strength of all four limbs, which was more remarkable on the lower limbs (3/5), normal deep tendon reflexes, and generalized myalgias on palpation.

On the second day of admission, when were unaware of her previous treatments (especially the neuroleptics), the patient presented a temperature of 40.5°C. Based on her clinical picture and follow-up with the family by social workers on her previous medications, the diagnosis of NMS was made. The patient was started on Orphenadrine (50mg PO 8 hourly), L-dopa and Carbidopa (125 mg orally eight hourly), and Bromocriptine (5 mg loading dose then 2.5 mg three times daily).

Haematological investigations were as follows: white cell count, 7.59 × 10 ^9^/L (normal range, 3.90–12.60 ×10 ^9^/L); ELISA test for HIV, positive; creatine phosphokinase, 1,467 U/L (20–180 U/L); Vitamin B12, 579 pmol/L (145–569 pmol/L); Toxoplasmosis ELISA, negative; VDRL, non-reactive; ferritin, 2136 ug/L (13–150 ug/L); iron, 2.2 µmol/L (9–30.4 µmol/L); transferrin, 1.32 g/L (2.5–3.8 g/L), percentage saturation, 10%; (15–50%) blood sugar, regular; hepatitis B surface Ag, negative; urea, 8.6 mmol/L (2.1–7.1 mmol/L); creatinine 112 µmol/L (49–90 µmol/L); alkaline COVID-19 test by PCR, negative; and CSF, average. Other blood test results (including CD4 and viral load) did not arrive during admission time. Computer tomography (CT) scan of the head was entirely normal.

There were no changes noted in her clinical condition during the period of her admission and on the third day of hospitalization, the patient died. No post-mortem examination was conducted due to wishes of the family.

## Discussion

### Epidemiology of NMS

Three previous studies have collected epidemiological data on NMS. One showed an incidence of 1.4% in 500 patients with one fatality recorded ^7^. This study reported that a delay in diagnosis often occurs. Another study recorded an incidence of 1.4/1000 over three years ^4^, the mortality rate was 14.28%, with 28% of patients developing NMS after antipsychotic medication. The third study conducted in Turkey from 1985 to 2005 reported on 36 patients, 22% of whom developed NMS after antipsychotic drug use ^8^.

### Risk factors 

It has been shown that age and sex of patients do not have much of a role in NMS development. No specific neuropsychiatric conditions can predispose one to NMS; however, more catatonia cases have shown a propensity to progress to NMS. Other systemic factors include agitation, restraint, and exhaustion. Metabolic factors such as dehydration and low serum iron have been shown to predispose to NMS. Drugs like dopamine antagonists and high potency antipsychotics have a higher risk associated than low potency and atypical anti psychotics ^9, 10^.

### Causative agents

The primary trigger causing NMS is dopamine receptor blockade, and the causative agent is an antipsychotic drug. The sudden cessation/reduction in the dosage of dopaminergic medications may also precipitate NMS ^11^. Other drugs that are not neuroleptic, but have antidopaminergic activity, have also been implicated in NMS's causality ( Table 1).

**Table 1.** Causative medications for neuroleptic malignant syndrome.

Neuroleptics	Typical	Haloperidol, Chlorpromazine, Fluphenazine
	Atypical	Clozapine, Risperidone, Quetiapine
Nonneuroleptic with antidopaminergic activity		Metoclopramide, Promethazine, Reserpine Lithium
Dopaminergic (withdrawal)		Levodopa, Amantadine

### Clinical presentation 

Clinical presentation of NMS usually has four so-called cardinal features, i.e. hyperthermia, muscular rigidity, autonomic instability, and altered consciousness level. However, many authors have reported more atypical presentations to this syndrome.

Hyperthermia of >38°C associated with profound diaphoresis is present in almost all cases reported and seems to be one of the most unifying factors in the different diagnostic criteria (discussed below) ^12^. Fever can be of late-onset and may increase fatality, as a diagnosis of NMS may not be made to the lack of fever. Rigidity is commonly present as lead pipe/cogwheel signs ^13^. However, it is also often associated with other neurological symptoms like tremors, sialorrhea, akinesia, dystonia, and dysphagia. Laboratory findings can help rule out other causes such as substance abuse and other systemic neurological or psychiatric conditions. Despite the following list being the most expected clinical presentation, as presented by the literature, none are specific to NMS ^11, 13^: elevated white cell count (leucocytosis); metabolic acidosis as shown by arterial blood gas and urea/electrolytes; elevated creatine kinase (CK); elevated serum muscle enzymes, e.g. lactic acid dehydrogenase and transaminases; elevated serum catecholamines, e.g. aldolase; and decreased serum iron levels. These presentations could lead to myoglobinuria and subsequent renal failure. CSF analysis in NMS patients is reported as normal in 95% of patients ^1^. Similarly, imaging, e.g. CT of the brain and magnetic resonance imaging, show unremarkable findings ^1^. Electroencephalogram shows features akin to those of metabolic encephalopathy ^1^.

### Diagnosis

Reaching NMS diagnosis is not easy, as much of the disease mimics many other conditions. However, the American Psychiatric Association Diagnostic and Statistics Manual of Mental Disorders (DSM-IV; Table 2) ^13^, the World Health Organisation International Classification of Diseases 10th revision (ICD-10), and the Caroff-Mann criteria from 1993 provide guidance. The most widely accepted diagnostic criteria is Levenson’s criteria, proposed in 1985, which has major and minor criteria for diagnosis; major criteria: fever, rigidity and elevated CK; minor criteria: labile blood pressure, tachypnoea, altered consciousness, and leucocytosis. All three of the major, or two major and four minor criteria must be met to diagnose NMS ^14^.

**Table 2.** Diagnostic and Statistical Manual of Mental Disorders-IV criteria for diagnosis of neuroleptic malignant syndrome.

A	Development of muscle rigidity and hyperthermia after exposure to neuroleptic medication.	
B	Two or more of the following:	
	1. Diaphoresis 2. Dysphagia 3. Tremor 4. Incontinence 5. Altered mental status 6. Mutism	7. Tachycardia 8. Elevated / labile blood pressure 9. Leukocytosis 10. Elevated Ck
C	Symptoms in A and B are not because of a substance, neurological, or general medical condition.	
D	Symptoms in A and B are not accounted for in mental condition.	

According to ICD-10, NMS is a fatal disease associated mainly with neuroleptic agents accompanied by dopaminergic receptor blockade in the lentiform nucleus, thalamus, head of caudate nucleus hypothalamus with autonomic dysregulation.

Caroff and Mann present the following criteria for NMS diagnosis ^15^: hyperthermia; muscle rigidity; use of neuroleptics within the last seven days; any five of altered mental status, tachypnoea, hypertension, tachycardia, hypotension, incontinence, diaphoresis, sialorrhea, elevated CK, myoglobinuria, or leucocytosis; absence of any other drug-induced, systemic, or neuropsychiatric disorder.

Though many authors and clinicians have often not found common ground in the diagnostic criteria, Gurrera *et al.* ^16^ showed that agreement was best between International Expert Consensus (IEC) criteria with a cut-off score of 74 and modified DSM-IV-TR criteria (sensitivity 69.6%, specificity 90.7%); this cut-off score demonstrated the highest agreement in all comparisons.

The sequelae of the condition usually happen after the initiation of neuroleptic drugs (or dopaminergic drugs). In about 16% of patients, NMS symptoms show within 24 hours; in another two thirds, symptoms show within one week; and almost all cases show symptoms at one month. Beyond a month, it is unlikely for the symptoms to start showing unless there has been an increase in the drugs' current dose ^1^.

### Differential diagnosis

Differential diagnosis can be broadly separated into four systemic causes: environmental, endocrine or infections, toxic/pharmaceutical agents, and psychiatric/neurological causes ^1, 11^. We discuss examples and distinguishing features in Table 3.

**Table 3.** Differential diagnosis and distinguishing features of neuroleptic malignant syndrome.

Differential diagnosis		Distinguishing features
***Systemic causes***		
*Environmental*	Heatstroke	History of exposure to heat
*Endocrine*	Pheochromocytoma Thyroid emergencies – thyrotoxicosis	Significantly elevated catecholamines and metanephrines Absence of neuroleptic medication
*Infections*	Sepsis Central nervous system infections, e.g. brain abscess, meningitis, encephalitis	Prodromal of viral illness Presence of seizures
*Toxins / Pharmacologic agents*		
Anaesthetics agents causing malignant hyperthermia Withdrawal from antiparkinsonian drugs, baclofen, alcohol Substance abuse – amphetamines, hallucinogens Serotonin syndrome		History of anaesthesia/surgery Positive toxicology/drug screen Presence of nausea, vomiting, diarrhea
*Psychiatric/Neurological causes*		
Extrapyramidal side effects Nonconvulsive status epilepticus Midbrain structural lesions.		Absence of fever or leucocytosis Presence of hyperkinesia Absence of neuroleptic treatment

### Pathophysiology

The actual pathophysiology of NMS is still not elucidated, but one hypothesis is that an antipsychotic-induced dopamine blockade does play a key role in triggering NMS ^17^. This is evidenced by the presentation of symptoms when initiating antipsychotics or stopping dopaminergic medications. Also, all reported drugs that have precipitated NMS have been dopamine receptor blocker medications. The decreased levels of homovanillic acid, a CSF metabolite of dopamine, also supports this theory ^18^. Other hypotheses are that sympathoadrenal dysfunction has a contributory role in NMS ^19^, or a low serum iron concentration can be a contributory factor in decreasing the number of dopaminergic receptors, thereby leaving patients susceptible to developing NMS. This is supported by the finding of low serum iron in patients with NMS ^20^. A final theory is that multiple neuroendocrine and neurochemical dysregulation cascade can lead to this NMS ^1^. Gurrera provides an excellent composite aggregation of the many postulated theories ^17^.

### Management

NMS should be considered an emergency by all managing physicians, and attending doctors should apply urgent treatment, even if the diagnosis is in doubt ^11^. There have been no systemic trials or studies conducted towards the best management protocol for the condition, mostly due to its rarity. As always, and as with any medical condition, the treatment is tailored to the clinical setting and the patient, but based on the many case reports, the following seem to be the most effective.

Primarily, cessation of the causative neuroleptic medication or if it is suspected that NMS is because of the withdrawal of a particular dopaminergic drug, then restart it as soon as possible. Supportive therapy, such as aggressive rehydration, management of hyperthermia and management of any complications (e.g. cardiopulmonary failure, seizures or arrhythmias) should be then initiated. Finally, as the severity of the condition worsens, empirical medication should be used ^1, 11^, as follows:

*Bromocriptine mesylate*: a dopamine agonist, is used to reverse hypodopaminergic. The starting dose of 2.5mg per os is eight to 12 hours and quantity is increased by 2.5mg every 24 hours to a maximum amount of 45mg/day. This should be used for at least ten days when the NMS has been caused by oral neuroleptics and about 2–3 weeks where depot preparations denote the cause.

*Dantrolene sodium:* a muscle relaxant that inhibits calcium release from the sarcoplasmic reticulum. Starting dosage is 1–2.5mg/kg iv bolus, then followed by 1mg/kg every 6 hours, to a maximum dose of 10mg/kg/day. Oral dantrolene (50–200mg/day) can be used in other less severe cases or when tapering down from the intravenous form. Dantrolene should be discontinued as soon as symptoms start to improve, as it is associated with a high risk of hepatic toxicity.

*Other dopaminergic agents:* the following have been reported in previous studies – amantadine hydrochloride ^21^, levodopa ^22^, and apomorphine ^23^.

*Benzodiazepines:* these have been used by clinicians to control agitation ^24^.

*Electro convulsant therapy:* a second-line treatment proposed for those patients not responding to empirical treatment ^25^.

There is always a challenge when restarting neuroleptic medications for patients that need continuous treatment, especially regarding NMS recurrence. The literature consensus is that the drug should be reinitiated at the lowest possible dose, with reasonable precautions taken and close monitoring after approx. two weeks of recovery from an NMS episode in oral neuroleptics and about six weeks for depot preparations. However, many authors opine the use of different neuroleptic medication; we must bear in mind that this is an idiosyncratic disease ^11^.

An algorithm adapted from Woodbury and Woodbury, which appears to be an invaluable tool in NMS management ^1, 26^.

### Complications 

There have been medical reports of cases where NMS has had atypical presentations and unexpected comorbidities ^12^. In one large study comprising of 1346 patients from 2002 to 2011, the authors report the commonest complication to be rhabdomyolysis (30.1%), acute kidney injury (17.7%), acute respiratory failure (16.1%), and sepsis (6.2%). Mortality rate was 5.6% ^27^. Hypoxemia and haemoconcentration that follow NMS predispose patients to cerebral infarction ^28^. The use of neuroleptics in trauma centers for burn victims has precipitated NMS ^29^. Complications can include dehydration, electrolyte imbalances, cardiac arrhythmias, aspiration pneumonia, myocardial infarction, deep venous thrombosis, and disseminated intravascular coagulation. Therefore, it is especially important to have close monitoring of patients.

A morphometric study of cardiac patients previously conducted reports neuroleptic cardiomyopathy (NCMP) and patients who died from NMS. The authors of this study described the damage in those patients’ myocardium due to an acute process that involved disturbances in microcirculation, interstitial edema, and dystrophic degenerative changes of cardiomyocytes ^30^. The severity of the myocardium damage in NMS is directly dependent on the NCMP, NMS, and HIV.

The incidence of NMS is high in HIV patients; the consensus is that it is so because of the changes that occur to the brain structure either from opportunistic infections secondary to HIV or to HIV itself. These patients have an increased possibility of developing NMS ^31^. However, it has not been easy to get a thorough picture of the epidemiology since many NMS symptoms also fall under symptomatology caused by other opportunistic infections ^32^. Nausea, vomiting, psychotic symptoms and agitation are all commonly seen in patients with HIV, and usually, many clinicians use neuroleptics to treat these symptoms ^33^.

Haloperidol is the most frequently associated medication with NMS. The main clinical signs are hyperthermia, rigidity, altered consciousness level, and autonomic disturbances. Hernández *et al.* present an algorithm adapted to manage patients with NMS (especially with extrapyramidal reactions) and HIV ^33^.

### NMS and COVID-19

There are scant publications about the presence of NMS and coronavirus disease 2019 (COVID-19). However, Soh *et al.* ^34^ have reported that two of their patients on the ventilator due to the COVID-19 developed delirium when they received benzodiazepine and neuroleptics. The patients subsequently developed NMS, which was managed and resolved. Kajani *et al.* ^35^ also reported a COVID-10 case with developed NMS, but the only causative agent they found was haloperidol given three weeks before onset. An autopsy showed a hyperaemic and oedematous brain. Therefore, it is unclear if COVID-19 itself can bring about changes that would lead to NMS due to changes it causes to the brain.

### Discussion of our case

Our patient meets the criteria by Levenson and the DSM-IV criteria for NMS ^36^. She developed NMS most likely due to the administration of antipsychotic medication and secondary to an idiosyncratic reaction. This occurred a few weeks after initiation, but it can occur even if the patient has been taking the drug for months to years ^37^. In our patient’s case, she had been on oral risperidone and haloperidol and the depot preparation of Clopixol for about eight weeks.

Our patient’s risk factors for developing NMS were the fact that she was on three different antipsychotic medications, she was HIV positive (new diagnosis, previously unknown), and had low serum iron levels in the blood, such a case has been reported by other authors ^38^. We want to highlight that our patient presented typical signs and symptoms for NMS and had elevated CK.

This case presented to us as we were dealing with the COVID-19 pandemic; and we were not aware of the psychiatric history at the time of admission, therefore a NMS diagnosis was made one day after admission. Social workers were recruited to track family members for a thorough history and medication details. The delay was not intentional, but the patient had what seemed like an atypical presentation. The atypical presentation of NMS was not evident immediately, as she presented with symptoms highly suggestive of COVID-19. Regarding other complications, our patient had a mild pre-renal injury, but blood results did not show the development of the other complications associated with NMS, as discussed above.

The recommendations for treatment of NMS currently are based on case reports and clinical experience as there is no published clinical trial to date. Therefore, NMS treatment of our patient followed the recommendations as discussed earlier: (1) stop causative agents; (2) aggressive supportive care (rehydration, treat hyperthermia, and other complications); and (3) specific medical therapy.

In our medical practice, we have treated a small group of HIV-positive women (N=5) with low CD4 count levels presenting with permanent focal simple motor seizures affecting the lower part of the face for a few days duration without other comorbidities followed by an unexpected death. We do not know the cause of death of these previously non-reported patients, but we believe that, to the best of our knowledge, it is the first time that NMS and this type of seizure in HIV patients has occurred in our region.

## Conclusions

We report a young HIV-positive female patient who died due to NMS preceded by the intake of typical/atypical neuroleptics. Apart from her altered consciousness, autonomic disturbances, extrapyramidal signs, and high fever, she also with presented permanent focal simple motor seizures on the lower hemiface. After reviewing the available medical literature, we found no similar case reported in the medical literature.

## Consent

Written informed consent for publication of this case report, along with any associated images, was obtained from the mother of the patient.

## Data availability

All data underlying the results are available as part of the article and no additional source data are required.
