# Supplementary material for: Case Report: Neuroleptic malignant syndrome in a HIV-positive patient
Source: F1000Res. 2021 Nov 4;9:1382. Originally published 2020 Nov 30. [Version 2] doi: 10.12688/f1000research.27165.2 (PMC9396113; doi:10.12688/f1000research.27165.2)
Supplement: Peer review history

# Peer review: Reviewer response for version 2

**DOI:** 10.5256/f1000research.56753.r146775

**Published:** 2022-08-22

**Authors:** Paroma Mitra

**Affiliations:** 1 New York University Grossman School of Medicine, New York City, NY, USA

The authors have brought up a novel case - an HIV positive patient with NMS (also with several antipsychotic medications - first generation). The patient's initial history and work up is very interesting and in the era of COVID-19. 

 The authors rightly bring this up given the diagnosis of HIV and likely more susceptibility to medications and opportunistic infections.

 What would have been interesting to delineate would have been - should lower doses of antipsychotic medication be used traditionally and if there is agitation should benzodiazepines be used more commonly?

 Another question is did the authors rule out NMDA encephalitis?

Are enough details provided of any physical examination and diagnostic tests, treatment given and outcomes?

Yes

Is the case presented with sufficient detail to be useful for other practitioners?

Yes

Is sufficient discussion included of the importance of the findings and their relevance to future understanding of disease processes, diagnosis or treatment?

Yes

Is the background of the case’s history and progression described in sufficient detail?

Yes

Reviewer Expertise:

Geriatric Psychiatry and Public Sector Psychiatry

I confirm that I have read this submission and believe that I have an appropriate level of expertise to confirm that it is of an acceptable scientific standard.

# Peer review: Reviewer response for version 1

**DOI:** 10.5256/f1000research.30009.r75587

**Published:** 2020-12-07

**Authors:** Ravindra Kumar Garg

**Affiliations:** 1 Department of Neurology, King George's Medical University, Lucknow, Uttar Pradesh, India

It is a nicely written case report. Though association of neuroleptic malignant syndrome (NMS) with HIV infection is not unique but topic has been adequately reviewed. Another point I want to make that possibly EEG was not done. I agree that many cases of neuroleptic malignant syndrome in COVID-19 has been described and authors have reviewed them as well.

Are enough details provided of any physical examination and diagnostic tests, treatment given and outcomes?

Yes

Is the case presented with sufficient detail to be useful for other practitioners?

Yes

Is sufficient discussion included of the importance of the findings and their relevance to future understanding of disease processes, diagnosis or treatment?

Yes

Is the background of the case’s history and progression described in sufficient detail?

Yes

Reviewer Expertise:

CNS infections

I confirm that I have read this submission and believe that I have an appropriate level of expertise to confirm that it is of an acceptable scientific standard.

# Response

**Published:** 2020-12-07

**Authors:** Humberto Foyaca-Sibat

**Affiliations:** Walter Sisulu University, South Africa

Dear Prof Ravindra Kumar Garg

 Thanks a lot for your revision; we highly appreciate your recommendations.

 It has been a great sense of delight to receive your very nice and professional comments.

 Kindest regards

 Prof. Foyaca MD. Ph.D
